# Significance of HPV16 Viral Load Testing in Anal Cancer

**DOI:** 10.1007/s12253-020-00801-7

**Published:** 2020-04-07

**Authors:** Ewa Małusecka, Ewa Chmielik, Rafał Suwiński, Monika Giglok, Dariusz Lange, Tomasz Rutkowski, Agnieszka M. Mazurek

**Affiliations:** 1 Center for Translational Research and Molecular Biology of Cancer, Maria Sklodowska-Curie National Research Institute of Oncology Gliwice Branch, 44-102 Gliwice, Poland; 2 Tumor Pathology Department, Maria Sklodowska-Curie National Research Institute of Oncology Gliwice Branch, Gliwice, Poland; 3 II Radiotherapy and Chemotherapy Clinic and Teaching Hospital, Maria Sklodowska-Curie National Research Institute of Oncology Gliwice Branch, Gliwice, Poland; 4 I Radiation and Clinical Oncology Department, Maria Sklodowska-Curie National Research Institute of Oncology Gliwice Branch, Gliwice, Poland

**Keywords:** HPV, HPV16 viral load, p16^INK4A^, p53

## Abstract

Human papilloma virus (HPV) is highly frequent among patients with anal squamous cell carcinoma, but the viral load (VL) differs between patients. This study aimed to compare the rate of HPV positivity, HPV16VL, p16^INK4A^ and p53 expression between treatment naive and recurrent anal cancer patients. HPV was genotyped via AmpliSens® HPV HCR-genotype-titre-FRT kit. HPV16 VL was determined via quantitative polymerase chain reaction-based in-house test. p16^INK4A^ and p53 expression was tested via immunohistochemistry. The cohort comprised 13 treatment-naive and 17 recurrent anal SCC patients. High-risk HPV was detected in 87% of cases, and HPV16 (73%) was the predominant genotype. The rate of HPV positivity was higher among women and nonsmokers, and majority of HPV-positive cases were also p16^INK4A^-positive. All p53-negative tumors were HPV16-positive. The most predominant p53 staining pattern in the HPV-positive group was scattered type, whereas it was diffuse type in the HPV-negative group. The HPV16 VL was higher in the treatment-naive group. Further, in the treatment-naive group, cases with scattered staining pattern of p53 had higher HPV16 VL than cases with diffuse staining pattern. The opposite result was noted in the recurrent cancer group. Moreover, p16-positive cases with scattered p53 staining pattern in the treatment naive group had higher HPV16 VL than their counterparts in the recurrent cancer group. In conclusion, the HPV VL, as is the association between VL and p16^INK4A^ /p53, is in an inversed trend in treatment naive and recurrent cancer patients, highlighting the importance of HPV VL measurement in anal SCC.

## Introduction

Anal squamous cell carcinoma (ASCC) is rare, accounting for only 0.5% of new cancer cases and a lifetime risk of only 0.2%. However, its incidence during the last 10 years has increased by 2.2% annually [1].The risk factors of ASCC include sex, race, smoking, human papilloma virus (HPV) and/or human immunodeficiency virus infection, certain types of sexual behavior, immunosuppression, and inflammatory diseases. Of these, HPV infection is currently the most frequently studied because approximately 90% of ASCC patients are HPV positive [2].

It is already known that HPV-positive and HPV-negative cancers are separate entities with respect to their molecular landscape and clinical behavior. HPV-positive tumors better respond to radiotherapy or chemoradiotherapy than their HPV-negative counterparts [3]. These differences can be partially explained by the diverse mutational spectra between groups defined by HPV status. HPV-dependent anal, cervical, and head and neck squamous cell cancer have been found to have similar patterns of molecular alterations [4–6]. Comprehensive analysis of mutations, copy number alterations, and rearrangements showed that HPV-negative cancers are characterized by *CDKN2A* and *TP53* gene mutations. Further, *PIK3CA* alterations, although more frequent in HPV-positive cases, are also highly common in the HPV-negative cohort.

Despite the high complete response rate (80%) in anal cancer patients, some remain resistant to chemoradiotherapy. To establish the molecular alterations behind these differences, a comparative genomic analysis of treatment-naive and recurrent tumor was performed [7]. Unexpectedly, the genomic alteration load was similar between treatment-naive tumors and chemoradiotherapy-treated recurrent tumors, and there were no differences in the frequency of genomic changes between the two groups [7]. However, the authors only described that 88% of cases were HPV16-positive, and they did not detail any differences in HPV status between the recurrent and the treatment-naive groups. Similar observations were reported by Mouw et al. [8], who studied matched pairs of primary-recurrent (post chemoradiotherapy) anal SCC cases via whole exome sequencing. Both primary and recurrent anal SCC harbored mutations also detected in other HPV-associated tumors. Moreover, the overall mutational burden was not significantly different between treatment naive and post-treatment tumors.

This study aimed to investigate whether the HPV16 viral load (HPV16 VL) can discriminate between treatment-naive and recurrent tumors. Additionally, we aimed to analyze the correlations between HPV VL and the expression of viral E6/E7 proteins, as well as cellular p53, p16^INK4A^ based on the finding that only a combination of HPV/p16^INK4A^-positivity corresponds to transcriptionally active HPV [9]. To the best of our knowledge, such an analysis has not been performed previously.

Because studies on HPV16 VL do not have an established cut-off value, their results cannot be directly compared [10]. Thus, although our study included a small number of cases, it allows for a direct comparison of VL values in treatment-naive and recurrent anal cancer.

## Patients and Methods

The study was performed using formalin-fixed, paraffin-embedded (FFPE) tissue samples obtained during diagnostic or therapeutic procedures from patients with ASCC. FFPE blocks were obtained from the archives of the Pathology Department of Maria Sklodowska-Curie National Research Institute of Oncology, Gliwice Branch. Patients were treated according to diagnosis and current disease status between 2006 and 2016. Data on age, sex, and tumor/nodal classification were collected from medical records. Smoking status was self-reported during admission.

### Detection of HPV in the Tumor Tissue

HPV DNA was isolated from FFPE tumor samples using the commercially available GeneMATRIX Tissue DNA Purification Kit (EUR_x_, Gdańsk, Poland). Depending on the tumor area, 3–10 consecutive tissue slices (5 μm) were used for DNA isolation. Macrodissection was performed, if necessary, to enrich tumor samples with cancer cells. For the quantitative polymerase chain reaction (PCR), 10 μL eluate containing 50 ng DNA (measured via NanoDrop) was used. HPV was genotyped using AmpliSens® HPV HCR-genotype-titre-FRT kit (InterLabService, Moscow, Russia), which enables detection and quantification of 14 high-risk HPV (hrHPV) strains (16, 18, 31, 33, 35, 39, 45, 51, 52, 56, 58, 59, 66, and 68). Positivity was defined as HPV Ct values below 30. To quantify HPV DNA viral load (copies/genome) in tissue samples we used real time PCR based on TaqMan technology. Amplification of TERT (human telomerase reverse transcriptase) was used as a marker of the total amount of genomic DNA presented in samples. The oligonucleotides (probe and primers) for HPV and TERT gene have been synthesized by Genomed S.A (Genomed S.A, Warsaw, Poland). Each measurement consisted 2 standard curves (genomic DNA and plasmid construct with HPV genome), negative control and a sample. All PCR reactions were performed using the Bio-Rad CFX96 qPCR instrument (Bio-Rad Laboratories, Hemel Hempstead, United Kingdom). Copies of TERT were converted to genome and viral load of HPV was expressed as the number of copies per genome.

### Immunohistochemistry Evaluation

The expression of viral E6 and E7, and p53 and p16^INK4A^ proteins was assessed via immunohistochemistry. Common conditions of immunohistochemical reaction for all antibodies were applied. Shortly, the slides were incubated overnight at 4 °C, then antigen was retrieved by boiling in EDTA buffer. Finally, horseradish peroxidase blocking (1% H_2_O_2_ in PBS) was employed prior to antibody incubation. ImmPRESS™ Universal Antibody, Polymer Detection Kit; Peroxidase (Vector Laboratories Inc., Burlingame, USA) with DAB as a chromogen was used for antibody detection. The following antibodies were employed: mouse monoclonal HPV16/HPV18 E6 Antibody (C1P5) (Novus Biologicals, Centennial, USA); mouse monoclonal HPV Type 16 E7 Antibody (8C9) (Thermo Fisher Scientific, Waltham, USA); monoclonal mouse anti-human p53 protein (DO-7) (DAKO/Agilent, Santa Clara, USA); and CINtec® p16 Histology (Ventana Medical Systems, Inc./Roche, Oro Valley, USA). Antibodies used for viral E6 and E7 immunohistochemical staining showed weak or very weak signal. Despite testing various conditions of immunodetection, high background staining was constantly observed. This precluded a reliable evaluation of immunoreaction, and therefore the results of E6 and E7 staining were excluded from further analysis. Positive p16^INK4A^ expression was defined as strong, diffuse nuclear, and cytoplasmic staining in ≥70% of cancer cells. This cut-off is consistent with previously published criteria developed by Singhi and Westra [11]. By contrast, p16^INK4A^ negativity was defined as absence of or faintly diffuse immunoreaction. p53 immunoreaction was assessed according to the staining pattern and number of positive cancer cells as follows: negative, 0% of cells are positive; scattered, less than 60% of cells are positive; and diffuse, more than 60% of cells are positive. p53 staining patterns were assessed according to the method proposed by Ando et al. [12]. Briefly, by comparing of TP53 mutations and p53 immunohistochemical analysis, they found that cases with scattered p53 staining pattern had wild type TP53 gene. Additional SNP-CGH array demonstrated that scattered-type tumors had no change in the structure of chromosome 17. Therefore, they concluded that tumors with p53 scattered-type staining may reflect a functionally active non-mutated TP53 gene. Similar results were reported by Kaserer et al. [13].

### Statistical Analysis

Chi-square test was used to evaluate the association between categorical variables. HPV16 VL was log_10_ transformed to achieve normal distribution. Two cases with coinfection of HPV16 and other hrHPV genotypes were excluded from the analysis. Continuous variables were analyzed via nonparametric Mann–Whitney U test. All statistical analyses were performed using Statistica software ver. 13.1 (Dell Inc., Tulsa, USA), and *p* < 0.050 was considered significant.

## Results

### Patient Characteristics

The cohort comprised 30 patients; of these, 13 were treatment-naive and 17 had recurrent ASCC. All patients in our cohort did not have distant metastases (M0), while 12 patients had lymph node metastases (one with N1, five with N2 and six with N3). hrHPV was detected in 26 cases (87%). HPV16 was found in 22 cases (73%), while other HPV subtypes (31, 33, 39, 45 and 52) were detected in 7 cases. Coinfection of HPV16 and other hrHPV subtypes was found in 2 cases. The incidence of hrHPV-positive cases was similar between the treatment-naive group and the recurrent cancer group. Three of the four hrHPV-negative cases belonged to the recurrent cancer group. The clinicodemographic data of the treatment naive and recurrent ASCC groups are presented in Table 1.

**Table 1:** Patient characteristics

	Total	Treatment-naive group	Recurrent ASCC group
Number of cases	30	13 (43%)	17 (57%)
Age (years), median (range)	67 (30.9–88)	60.6 (46.8–88)	71 (30.8–81)
Sex			
Male	8	3 (37%)	5 (63%)
Female	22	10 (45%)	12 (54%)
Tumor classification			
T1	3	2 (67%)	1 (33%)
T2	5	2 (40%)	3 (60%)
T3	13	6 (46%)	7 (54%)
T4	9	3 (33%)	6 (67%)
Nodal status			
N0	18	8 (44%)	10 (56%)
N1	1	1 (100%)	
N2	5	1 (20%)	4 (80%)
N3	6	3 (50%)	3 (50%)
Smoking status^a^			
Nonsmoker	11	4 (36%)	7 (64%)
Ever smoker	16	9 (56%)	7 (44%)

^a^Data on smoking status were available only in 27 cases

### Human Papilloma Virus Viral Load and Positivity

The log_10_ transformed HPV16 VL ranged from 0.628 to 3.77 with median 2.12. In 2 cases co-infection of HPV16 (VL = 2.13 and 2.67) with HPV45 (one case) and HPV52 (one case) was found, both in trace amounts. In 3 HPV16-negative cases, the log_10_ transformed HPV31 VL, HPV33 VL and HPV39 VL were 1.71, 1.73 and 1.89, respectively. In 2 other HPV16-negative cases, trace amounts of HPV33 DNA and HPV45 DNA were found. Comparison between HPV16 and other types did not show statistically significant differences in VL levels (*p* = 0.819). However, it was observed that VL values of the other types were within the lower quartile of HPV16 VL.

HPV16 VL was higher in the treatment-naive group than that in the recurrent cancer group, but the difference was not statistically significant. Associations between clinical and molecular data with respect to HPV16 positivity/VL in the two groups are shown in Tables 2 and 3. Majority of patients in both groups were HPV-positive women. Further, in both groups, women had higher HPV16 VL than men, but this difference was not statistically significant.

**Table 2:** Clinico-pathological and molecular features in the treatment-naive group

	HPV-positive	HPV-negative	*p* value (*Χ*^2^ test)	HPV16 viral load (log_10_ transformed)	p value (Mann-Whitney U test)
Age (years), median (range)	64.3 (46.8–88)	60 (59.2–69.1)		2.13	
Sex					
Male	2 (67%)	1 (33%)		1.87	
Female	8 (80%)	2 (20%)	*p* = 0.63	2.14	*p* = 0.89
Tumor classification					
T1 + T2	2 (50%)	2 (50%)		1.93	
T3 + T4	8 (89%)	1 (11%)	*p* = 0.12	2.14	*p* = 1.0
Lymph node involvement					
Negative	6 (75%)	2 (25%)		2.21	
Positive	4 (80%)	1 (20%)	*p* = 0.84	2.14	*p* = 0.74
Smoking status^a^					
Nonsmoker	4 (100%)	0		3.04	
Ever smoker	6 (67%)	3 (33%)	*p* = 0.18	1.14	p = 0.03
p16 expression					
Negative	1 (50%)	1 (50%)		1.14	
Positive	9 (82%)	2 (18%)	*p* = 0.32	2.22	p = 1.0
p53 staining pattern					
Negative	2 (100%)	0		1.63	
Scattered	4 (100%)	0		2.63	
Diffuse	4 (57%)	3 (43%)	p = 0.18 (negative vs. scattered vs. diffuse)	0.76	*p* = 0.22 (Kruskal-Wallis test) (negative vs. scattered vs. diffuse)
Smoking history (pack-years^b^), median (N)	25.5 pack-years (4)	15.0 pack-years (3)			*p* = 0.85

^a^Data on smoking were available in only 27 cases, while data on smoking duration and number of cigarettes smoked were available for 13/16 smokers

^b^Pack-year is calculated by multiplying the number of packs of cigarettes smoked per day by the number of years the person has smoked

**Table 3:** Clinico-pathological and molecular features in the recurrent ASCC group

	HPV-positive	HPV-negative	*p* value (*Χ*^2^ test)	HPV16 viral load (log_10_ transformed)	*p* value (Mann-Whitney U test)
Age (years), median (range)	65.5 years (30.8–81)	71 years (61–77.2)		1.56	
Sex					
Male	1 (20%)	4 (80%)		1.19	
Female	11 (92%)	1 (8%)	*p* = 0.003	1.61	*p* = 1.0
Tumor classification					
T1 + T2	1 (25%)	3 (75%)		1.11	
T3 + T4	11 (85%)	2 (15%)	*p* = 0.02	1.61	*p* = 1.0
Lymph node involvement					
Negative	6 (60%)	4 (40%)		2.17	
Positive	6 (86%)	1 (14%)	*p* = 0.25	1.23	*p* = 0.12
Smoking status^a^					
Nonsmoker	6 (86%)	1 (14%)		1.61	
Ever smoker	4 (57%)	3 (43%)	*p* = 0.23	2.67	*p* = 1.0
p16 expression					
Negative	2 (33%)	4 (67%)		2.5	
Positive	10 (91%)	1 (9%)	*p* = 0.01	1.55	*p* = 0.55
p53 staining pattern					
Negative	2 (100%)	0		1.67	
Scattered	8 (80%)	2 (20%)		1.43	
Diffuse	2 (40%)	3 (60%)	*p* = 0.17 (negative vs. scattered vs. diffuse)	2.22	*p* = 0.82 (Kruskal-Wallis test) (negative vs. scattered vs. diffuse)
Smoking history (pack-years^b^), median (N)	20.74 pack-years (4)	6.7 pack-years (2)			*p* = 0.48

^a^Data on smoking were available for only 27 cases, and data on smoking duration and number of cigarettes smoked were available for 13/16 smokers

^b^Pack-year is calculated by multiplying the number of packs of cigarettes smoked per day by the number of years the person has smoked

Analysis according to smoking status showed that the rate of HPV16-positivity was higher in nonsmokers than that in smokers (91% vs 63%; *p* = 0.09). The correlation between smoking and HPV16 was similar in both groups. However, a comparison of HPV16 VL in these groups showed marked differences. In the treatment-naive group, nonsmokers had significantly higher HPV16 VL than smokers (*p* = 0.03). Meanwhile, the opposite finding was found in the recurrent cancer group, that is, smokers had higher HPV16 VL than nonsmokers.

Advanced-stage tumors (T3/T4) were more frequently HPV positive than early stage tumors (T1/T2) (*p* = 0.007). The rate of lymph node involvement was also higher in HPV-positive, advanced tumors than their HPV-negative counterparts (*p* = 0.08). Meanwhile, the correlation between HPV positivity and lymph node status was not significantly different between advanced-stage and early-stage tumors. Advanced tumors (T3/T4) also had higher HPV16 VL than T1/T2 tumors, but the difference was not statistically significant.

### Relationship between HPV and p53 Expression

**Fig. 1:**
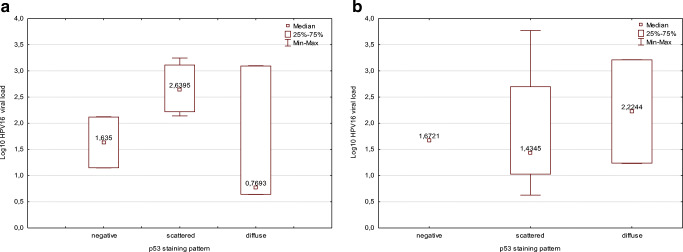
Comparison of HPV16 VL according to p53 staining patterns in the treatment-naive (**a**) and the recurrent (**b**) group

The patterns of p53 staining were categorized into three as negative, scattered, and diffuse. In total, 4, 14, and 12 cases showed negative, scattered, and diffuse patterns, respectively. All four p53-negative tumors were HPV16 positive. With respect to staining pattern of p53, the most predominant was scattered pattern in the HPV-positive group, whereas it was diffuse pattern in the HPV-negative group (*p* = 0.05). With respect to the correlation between HPV16 VL and p53 pattern, cases with scattered staining pattern in the treatment-naive group had higher HPV16 VL than cases with diffuse staining. By contrast, cases with diffuse staining pattern had higher HPV16 VL than those with scattered staining pattern in the recurrent cancer group (Fig. 1).

There was no statistically significant relationship between p53 staining pattern and sex or age in both groups. Meanwhile, with respect to staining pattern according to smoking status, contrasting trends were noted. The predominant p53 staining pattern was diffuse among smokers in the treatment-naive group, whereas it was scattered among nonsmokers in the recurrent cancer group. All p53-negative tumors were advanced-stage tumors (T3/T4). There was no correlation between nodal status and p53 expression in both groups.

### Relationship between HPV and p16^INK4A^ Expression

In total, 22/30 cases were p16^INK4A^ positive. Women were more frequently p16 positive than men in both groups, but the difference did not reach statistical significance (*p* = 0.08). However, although p16^INK4A^ was significantly correlated with HPV16 (*p* = 0.007) (Table 4), not all HPV16 positive cases were also p16 positive.

**Table 4:** p16^INK4A^ positivity vs. HPV16 positivity

	HPV16 negative	HPV16 positive	Total
p16-negative	5 (63%)	3 (37%)	8 (27%)
p16-positive	3 (14%)	19 (86%)	22 (73%)
Total	8 (27%)	22 (73%)	

In Table 4 correlation between p16 and HPV16 for whole cohort is shown. Considering separately treatment-naive and recurrent cancer groups these association was similar, except for cases p16-negative/HPV16-negative, which were more frequent in recurrent cancer group (4/5 cases).

p16-positive cases had higher HPV16 VL than p16-negative cases, without statistical significance. Further, p16-positive cases with above median HPV16 VL were predominant in the treatment-naive group, whereas p16-positive cases with below median HPV16 VL were predominant in the recurrent cancer group (*p* = 0.04).

In total, 19 cases were p16+/HPV+ (Table 4). Scattered p53 staining pattern was more frequent in these cases (presumed HPV transcriptional activity) than in cases with other combinations of p16/HPV status, in which diffuse pattern of p53 staining predominated (*p* = 0.05). This trend was observed in both groups. p16-positive cases with scattered p53 staining pattern in the treatment-naive group had significantly higher HPV16 VL than their counterparts in the recurrent cancer group (*p* = 0.07).

## Discussion

Studies have shown the importance of HPV VL testing for stratifying HPV-positive patients. High VL was found to be a favorable prognostic factor in various HPV-related cancers, including cervical cancer [14], anal cancer [15], and head and neck cancer [16]. However, there have been concerns on the accuracy of VL analysis. For example, the measurement of HPV VL is influenced by the choice of method of detection (hybridization vs. PCR) and targeted HPV region [17]. This implies a wide range of absolute values, and marked differences independent of method of VL evaluation have been found within single studies. The level of HPV16 VL observed by us in the anal cancer tissues (median 2.12; l.q 1,17 - u.q. 2.85) (median 134, l.q. 15 - u.q. 754 copies/genome) was comparable to the level of HPV16 VL in the oropharyngeal cancer tissues (median 2.27; l.q. 1.61 - u.q. 2.99) (median 190, l.q. 40 - u.q. 992 copies/genome) [18]. The HPV VL of other types was slightly lower than the HPV16 VL, though, without statistical significance. Although it is risky to draw conclusions about the lower VL of other types based on a small group of patients, one of the studies carried out on cervical samples showed that HPV16 is distinguished by the highest VL (followed by HPV51 and HPV45) [19]. Another work shows that HPV18 has a lower VL than HPV16, while the highest VL was observed for HPV58 [20]. The authors note that type-specific viral load level can be a useful diagnostic biomarker, but these conclusions require confirmation on a larger group.

In our study, we directly compared HPV16 VL in treatment-naive and recurrent ASCC. We found that HPV16 VL was higher in treatment-naive than in recurrent cancer, but the difference was not statistically significant. As we were not able to find any study comparing HPV16 VL between treatment naive and recurrent anal cancer, we referred to a study on primary and recurrent HPV-dependent vulvar cancer [21]. There were no differences in HPV16 VL between primary vulvar squamous cell cancer and matched local recurrences and/or metastases. Although VL somewhat varied between time points, the results in individual patients were relatively constant. The authors concluded that time elapsed and new milieu (lymph node or metastasis) seem to have no influence on VL [21]. The patients enrolled in that study were treated with surgery, and therefore the influence of treatment was negligible. As patients in the current cohort were treated with chemoradiotherapy, we believe that our study can be better compared to that by Hu et al. [22] in which HPV VL was used as outcome measure for determining the efficacy of photodynamic therapy for genital warts. HPV VL level significantly diminished during therapy, indicating treatment effectiveness. Moreover, Badaracco et al. [23] reported that HPV DNA clearance was associated with better patient outcomes in cervix carcinoma patients treated with chemoradiotherapy, providing evidence that HPV DNA detection is a valuable tool for assessing treatment efficacy. Collectively, the findings reported by Badaracco et al. [23] and Hu et al. [22] and those in the current study show that diminished HPV16 VL in recurrent ASCC indicates chemoradiotherapy-dependent decrease of VL level.

Other well established factors influencing HPV VL level are sex and smoking. The association between sex hormones and HPV is best documented in cervical diseases. Epidemiological studies have shown that oral contraceptives and multiple pregnancies are risk factors for cervical cancer [24]. Further, HPV-positive women with high estradiol levels had higher risk of cervical cancer than women who were only either HPV-positive or exhibited high levels of estradiol [25]. These results confirm the additive effect of estradiol and HPV infection found in experimental studies. Cervical and foreskin keratinocytes immortalized with HPV16 showed enhanced 16α-hydroxylation of estradiol and increased proliferation than normal cells [26]. Another study showed that estradiol and 16α-hydroxyestrone increased the number of proliferating cells and cause anchorage-independent growth of HPV immortalized keratinocytes [27]. While data on the association between HPV and hormones are abundant, data on HPV VL are limited. A study on the influence of pregnancy on HPV VL reported no differences in viral copy number/cell between non-pregnant and pregnant women [28].

Another factor associated with HPV VL is smoking. Smoking among HPV-positive women has been shown to be among the precipitating factors increasing the risk of cervical cancer. The chemical components of tobacco and its metabolites have been found in the cervical mucus of active and passive smokers [29]. Moreover, levels of nicotine and its metabolites correlated with smoking intensity [30]. Experimental studies showed that HPV-positive keratinocytes transiently exposed to benzo[a]pyrene demonstrated enhanced proliferation capacity without tumor formation in nude mice, whereas cells modified with chronic benzo[a]pyrene exposure demonstrated a malignant phenotype in organotypic “raft” culture and developed tumors in nude mice [31]. Another aspect of the relationship between HPV and smoking was investigated by Wei et al. [29] who analyzed the effect of mainstream tobacco smoke condensate (MSTS-C) on cervical cells with either episomal or integrated HPV form. MSTS-C exposure led to increased replication of viral genome and transcription of the early genes in cells with episomal hrHPV, but not in cells with integrated hrHPV. Consistent with increased E6 transcription, decreased p53 protein levels were found. Loss of p53 activity in HPV episomal cells resulted in higher levels of double-strand breaks and mutation rate, but apoptosis was not activated as compared to cells containing integrated HPV. These data show that tobacco smoke is a cofactor in HPV-related cancers dependent of the HPV episomal/integration status.

In anal cancer no association between HPV16 VL and smoking was reported [15]. By contrast, a comparison of HPV16/18 VL in cervical smears from women with no detectable abnormality showed that current smokers had significantly higher HPV16 VL or HPV18 VL than never smokers [32]. These results are in contrast to our findings in which HPV16 VL was higher in nonsmokers than that in smokers. We also previously found that HPV16 VL was higher in nonsmokers in HPV-dependent oropharyngeal cancer [18].

Of interest is the relationship between p53 staining pattern and HPV. In this study, we analyzed p53 staining pattern following the classification described by Ando et al. [12], who reported that scattered pattern corresponded to wild type TP53 and diffuse pattern to abnormal TP53. A similar classification was applied by Kaserer et al. [13] by studying colorectal adenocarcinomas. Although they found no perfect match between immunohistochemistry and TP53 gene analysis, they concluded that diffuse p53 staining pattern represents functional inactivation of the protein, regardless of gene alteration. By contrast, an analysis of HPV and p53 via immunohistochemistry and gene sequencing in HPV-negative anal cancer showed that the frequency of p53-positive cells varied significantly [33]. As we did not perform TP53 gene analysis, the relationship between HPV and *TP53* mutational status was not analyzed. However, in the current study, scattered staining pattern was predominant in the HPV-positive cohort, while it was diffuse staining pattern in the HPV-negative cohort. This observation indirectly confirms the findings reported by Meulendijks et al. [33] that the HPV status does not correspond to the definite number of p53-positive cells/staining pattern. However, we did not identify any mechanism that can explain the inversed correlation between HPV16 VL and p53 pattern and other variables in treatment-naive and recurrent cancer, probably because of the small number of samples analyzed. Further large-scale studies are needed.

## Conclusions

Considering that HPV16 VL is a valuable prognostic marker in HPV-dependent SCC cancers, its measurement should be standardized. Moreover, given that treatment naive and recurrent cancer showed opposite/inversed characteristics, including the association between viral load and p16^INK4A^/p53, further characterization of samples not only with respect to HPV positivity, but also to VL will provide novel information on the mechanisms of cancer recurrence.
